# New perspectives on preshearing history in granular soils

**DOI:** 10.1038/s41598-023-31419-9

**Published:** 2023-03-20

**Authors:** L. Knittel, M. Tafili, C. E. Grandas Tavera, Th. Triantafyllidis

**Affiliations:** 1 Keller Grundbau GmbH, Renchen, Germany; 2 Foundation Engineering and Environmental Geotechnics, Ruhr-University Bochum, Bochum, Germany; 3 Soil Mechanics and Foundations/Geotechnical Engineering, Brandenburg University of Technology, Cottbus, Germany

**Keywords:** Civil engineering, Natural hazards, Coal, Coarse-grained models, Computational methods

## Abstract

The design of deep dump slopes for opencast mines usually requires information about the soil resistance to liquefaction during earthquakes. This resistance depends not only on the initial stress, the initial density, and the amplitude of the cyclic loading, but also on the preshearing, that is, the deviatoric stress path applied to the soil before the cyclic loading. To explore the influence of preshearing on the subsequent soil behaviour, a set of triaxial tests with a combination of undrained preshearing and drained stress cycles using two sample preparation methods is presented. It is shown that the preshearing as well as the preparation method have a major influence on the strain accumulation upon cyclic loading. Simulations of the experiments with four advanced constitutive models reveal that neither the long-lasting effect of preshearing nor the preparation method can adequately be captured by all of the models. This deficiency of the constitutive models can lead to unsafe designs due to the overestimation of the cyclic resistance to liquefaction and to the underestimation of long term settlements.

## Introduction

**Figure 1:**
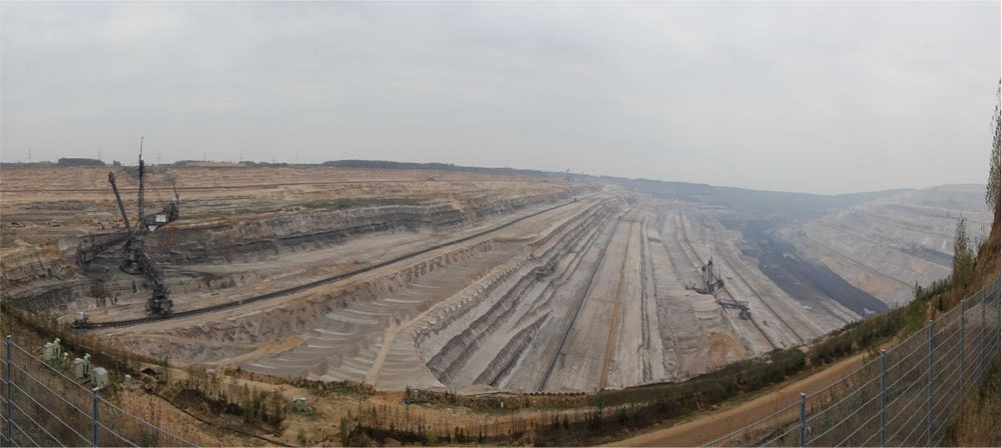
View of the extraction side of the lignite opencast mine Hambach (left hand side) with a surface of 85 km$^2$ and depth 400 m created with coal excavator and dumping site (right hand side)^1^.

Slope stability and long-term settlements assessments belong to the most challenging aspects of the design of dump slopes for opencast lignite mines. This holds especially for deep mines, like Hambach (Germany), where the loosely dumped granular layers may reach a depth of 400 m and the re-cultivation of the area after the lignite extraction is intended, see Fig. 1. To prevent catastrophic events, the design of dump slopes requires information about the soil resistance to liquefaction during possible earthquakes. But even if the liquefaction does not take place, excessive accumulation of settlements due to cyclic and/or quasi-static loading during and after the groundwater flooding can endanger the re-cultivation processes. Of note, an earthquake (undrained shearing) can present the preshearing of the next seismic loading. Adequate predictions of the liquefaction and the stress and strain accumulation upon cyclic loading, including the influence of density and deposition method on the soil behaviour are therefore essential.

With regard to liquefaction, the influence of density, consolidation stress as well as the amplitude of cyclic loading have been widely studied over the last decades. In general, undrained triaxial tests on samples consolidated under isotropic direction show that dense samples require more cycles of the applied load to reach liquefaction than loose ones. However, this is not true when the directions of preloading and subsequent loading differ.

**Figure 2:**
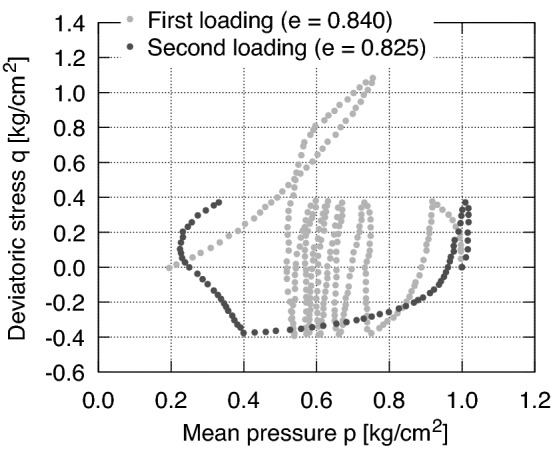
Behaviour of sand subjected to large preshearing on Fuji river sand by Ishihara and Okada^2^ (digitized data).

Using undrained triaxial tests, Ishihara and Okada^2^ studied the influence of the loading history (preloading) on the liquefaction resistance of Fuji river sand. They interpreted the preloading as either precompression or preshearing. In case of precompression the soil experienced an isotropic compression stress greater than that at the beginning of the subsequent shearing. In the event of preshearing the soil experienced a given deviatoric stress before the subsequent loading. By increasing the stress ratio from the isotropic stress axis during preshearing, they observed a tendency of the sample to contract while relatively small shear strains developed. Under drained conditions contraction results in an increase of volumetric strain, while under undrained shearing it renders an increase of excess pore water pressure. Further increase of the stress ratio led, in contrast, to dilation and to much larger shear strains. Under drained or undrained conditions dilation results in a decrease of volumetric strain or excess pore water pressure (increase of mean effective stress), respectively. In^2^ as well as widely in the geotechnical literature the stress ratio at which the soil behaviour changes from contraction to dilation is denoted as *the phase transformation line* (PTL). Accordingly, loading histories reaching stress ratios smaller than PTL were termed *small preshearing* whereas those going beyond the PTL were called *large preshearing*. Fig. 2 (digitized from^2^) shows the behaviour of Fuji river sand subjected to large preshearing with subsequent cyclic undrained loading. After some cycles with a deviatoric stress amplitude of $q^{{\text{ ampl }}}=0.4$ kg/cm$^2$ (first loading), the sample was loaded beyond the PTL (large preshearing) with a deviatoric stress of $q\approx 1.1$ kg/cm$^2$. Then, the resulting excess of pore water pressure was dissipated by opening the drainage until the initial isotropic effective stress (*p* = 1.0 kg/cm$^2$) was recovered (re-consolidation). Finally, the sample was subjected to undrained cycles of deviatoric stress (second loading) with the same amplitude as on the first loading. The experiment shows that the effective stress reduces faster with the number of load cycles for the case of large preshearing (second loading) than for the case without preshearing (first loading). Even though the void ratio before second loading ($e=0.825$) is lower than the one prior to first loading ($e=0.840$) the denser state subjected to same loading amplitude liquefies easier. Therefore, the loading history (preloading) plays a major role (sometimes even more significant than density) in the material behaviour and can reduce its resistance against liquefaction significantly.

Several studies in the literature^2–7^ have demonstrated that a preloading history composed of drained or undrained cycles with small strain amplitudes (say less than 1%) usually increases the liquefaction resistance during a second undrained phase following re-consolidation (similar observations were made in drained cyclic tests regarding a reduction of the settlement rate^8^). In contrast, a considerable reduction of the undrained cyclic strength may be caused by a preceding cyclic mobility involving large strain amplitudes or by a drained monotonic preloading accompanied by dilatancy^2–4^. Such reduction is sometimes also observed in situ, when a re-liquefaction of the sand occurs during an after-shock event with smaller intensity than the main earthquake^9^. Other aspects of preloading history in dependence on sample preparation techniques are discussed in^10–20^, indicating that the re-liquefaction resistance of sand is much more sensitive to the microstructure formed by consolidation after loading in the early stage than its quasi static strength^9,21^. Oda el al. (2001) concluded based on investigations of Toyoura sand samples, that the inherent anisotropy, hence preferred orientation of contact normals is one of the most important factors in controlling the liquefaction resistance. The inherent anisotropy is, however, easily altered in the subsequent shearing process, and new anisotropy (induced anisotropy) is produced^22^. The induced anisotropy by a columnlike structure and connected voids, both of which grow parallel to the major principal stress direction, is responsible for the drastic reduction in liquefaction resistance by preshearing. The connected voids between neighboring columns are easily closed when they are first stressed perpendicular to their elongation direction, which causes the large volume contraction under drained conditions and the rapid increase of excess pore-water pressure under undrained conditions^9^. The shape of the voids, as well as their sizes, is of particular importance to evaluate the liquefaction resistance of presheared sand^9^. Furthermore^23^, encountered the spatial and temporal distribution of fabric, contacts between the grains and voids throughout one Hostun sand sample even at the initial state heterogeneous.

In contrast to the well-studied liquefaction phenomenon and despite its importance for the design of re-cultivation projects, the influence of small and large preshearing on the strain accumulation upon cyclic loading has received less attention so far. In this paper, the strain development along a prescribed stress path with varying stress ratios for different loading histories is investigated. The tests were conducted on a triaxial testing device with independent control of the vertical and the horizontal stresses. The loading histories include large and small preshearing in both triaxial compression and extension. The results of the experiments were compared with numerical simulations of four advanced constitutive models for sand to examine their ability to track the loading history. Effects of sample preparation techniques were also addressed.

The paper is structured as follows: chapter two describes the properties of the Karlsruhe fine sand, the details of the triaxial device, and the preparation of the samples. Chapter three shows the resulting strain paths for different loading histories, while in chapter four the experiments are compared with the numerical simulations. Simulations of the experiments of Ishihara and Okada^2^ are also included. After the conclusions in chapter five, the constitutive models are briefly described in four appendices.

## Symbols and notation

The notation of this article is specified as follow: vectors and second-order tensors are denoted by bold symbols, e.g. the effective Cauchy stress ${{\varvec{\sigma }}}$ and strain tensor ${{\varvec{\varepsilon }}}$. Bold calligraphic letters denote fourth order tensors (e.g. $\pmb {\mathcal {M}}$). Tensor operations are used following the Einstein summation convention. Continuum mechanics conventions are followed, i.e. compression is defined negative.

$||\textbf{X}||=\sqrt{{\rm tr}\,\textbf{X}^2}$ is the Frobenius norm of $\textbf{X}$, whereby ${\rm tr}\,\textbf{X}$ is the sum of the diagonal components of $\textbf{X}$. $\mathring{{{\varvec{\sigma }}}}$ is the co-rotational, objective stress rate. The stretching tensor ${{\varvec{\varepsilon }}}$ is the symmetric part of the velocity gradient. The void ratio *e* is the ratio of the volume of the voids $V_v$ to the volume of the solids $V_s$. $p=-1/3{~\rm tr}\,{{\varvec{\sigma }}}$ is the mean effective stress, $\varepsilon _{\text{ v }}={\rm tr}\,{{\varvec{\varepsilon }}}$ is the volumetric strain. When dealing with axisymmetric conditions, the Rendulic plane is used for illustrative purposes in geotechnics. Thereby the axial stress is denoted with $\sigma _1'$ and the radial stress with $\sigma _2' (=\sigma _3')$ and the respective strains are $\varepsilon _1$ and $\varepsilon _2=\varepsilon _3$. The remaining Roscoe invariants for triaxial conditions are defined as $q=-(\sigma _1-\sigma _2)$ and $\varepsilon _q=-2/3\,(\varepsilon _1-\varepsilon _2)$. Initial values are labeled with the subscript $\sqcup _0$.

The isometric variables $P = \sqrt{3} p$ and $Q = \sqrt{3/2}\, q$^24^ are advantageous in connection with studies on the influence of cyclic loading because the lengths of the stress paths and the angles between two polarizations are preserved when transferred from a principal stress coordinate system to the *P*-*Q* plane, in contrast to the *p*-*q* representation. In the strain space, the belonging isometric strain variables are $\varepsilon _P = \varepsilon _v/\sqrt{3}$ and $\varepsilon _Q = \sqrt{3/2}\, \varepsilon _q$.

## Device, material and preparation

**Figure 3:**
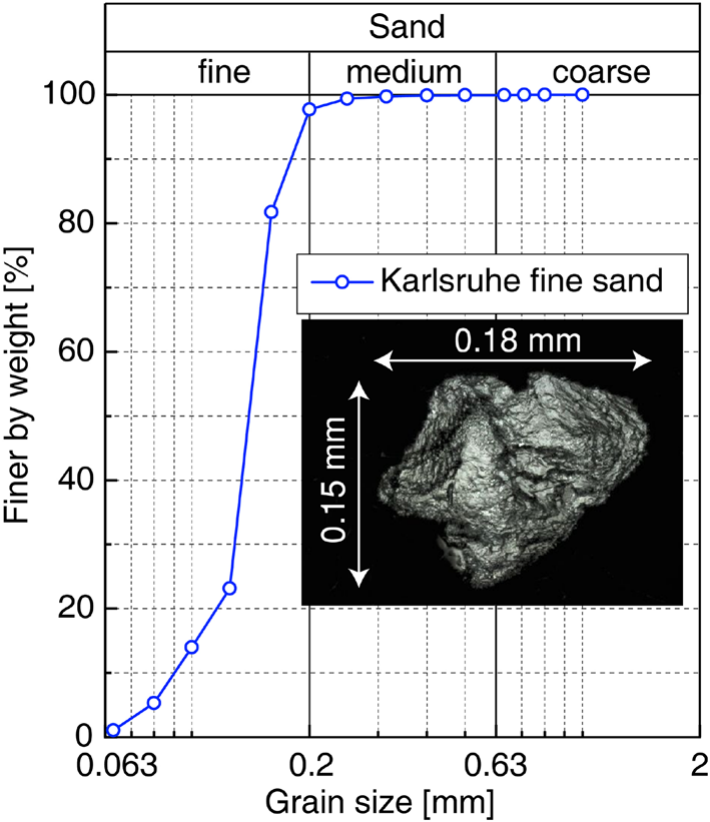
Grain size distribution curve after^27^ of the used KFS and microscopic image of a grain out of^28^.

The uniform ,,Karlsruhe fine sand” (KFS, mean grain size $d_{50}$ = 0.14 mm, uniformity coefficient $C_u = d_{60}/d_{10}$ = 1.5, minimum void ratio $e_{\min }$ = 0.677 and maximum void ratio $e_{\max }$ = 1.054^25,26^, grain density $\varrho _{s}$ = 2.65 g/cm$^3$, subangular grain shape) has been used in the experiments. The grain size distribution curve and a microscopic image of the grains is shown in Fig. 3.

**Figure 4:**
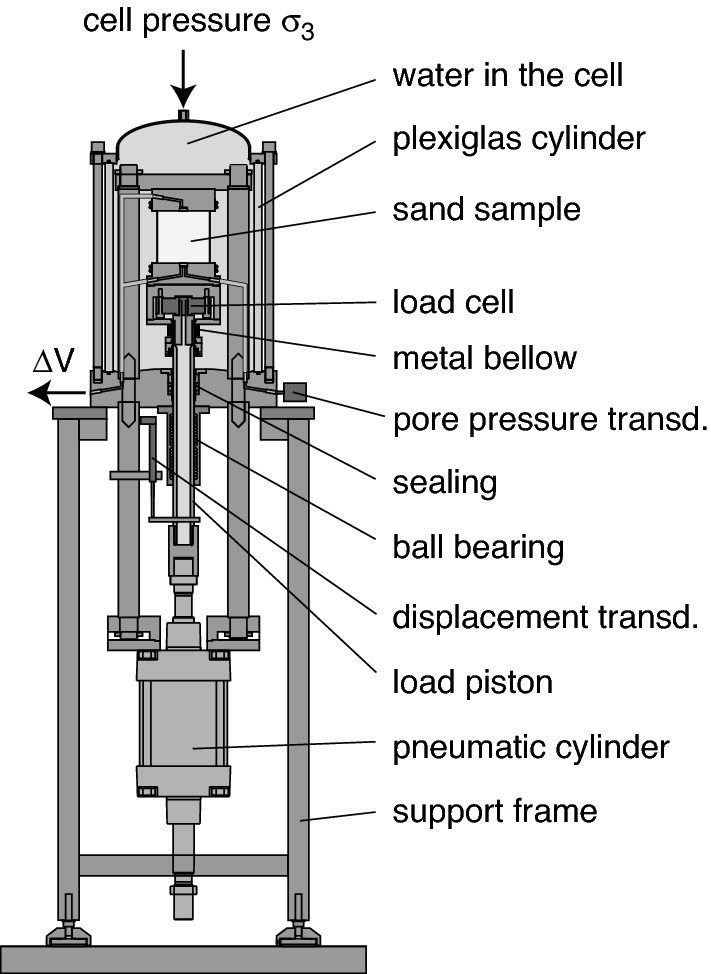
Scheme of triaxial device for cyclic tests^31,32^.

A scheme of the triaxial device which was used for all triaxial tests is shown in Fig. 4. In this device the cyclic vertical loading is applied from the bottom using a pneumatic loading system. The vertical load is measured at a load cell located directly below the sample base plate. For the cyclic loading of the lateral stress, another pneumatic loading system was connected to the cell volume. Vertical displacement is measured with a displacement transducer with an accuracy and resolution of 10 $\upmu$m mounted to the load piston. System deformation has been carefully determined in preliminary tests on a steel dummy and subtracted from the measured displacements. The samples were tested fully water-saturated and volume changes were obtained from the squeezed out or sucked in pore water using a system of two burettes (one connected to the drainage lines, one with constant water level) and a differential pressure transducer. The end plates were equipped with small central porous stones (diameter 15 mm). The friction at the end plates was reduced by smearing the end plates with a thin layer of grease followed by a latex rubber disk of 0.4 mm thickness. Latex membranes of 0.4 mm thickness were used to surround the sample. The application of the stress paths necessitates the cyclic variation of cell pressure, which can lead to membrane penetration effects^29,30^. These were found to be negligible for $d_{50}$ = 0.14 mm^1^.

**Figure 5:**
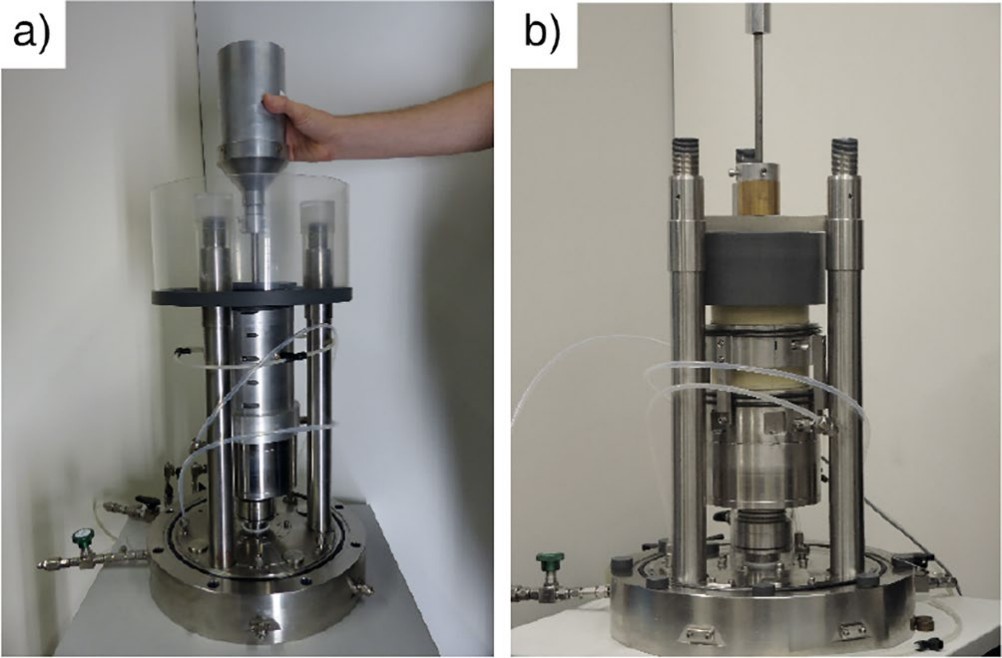
Preparation of the sample by (**a**) dry air pluviation method (AP) and (**b**) moist tamping technique (MT)^34^.

The full cylinder samples, with diameter *d* = 100 mm and height *h* = 200 mm, were prepared using either the dry air pluviation (AP, Fig. 5a) or the moist tamping method (MT, Fig. 5b). The combination of specimens with *h*/$d = 2$ and greased end plates results in a more homogeneous distribution of the void ratio at the states investigated here close to the limit state. The AP-method is carried out by manual trickling out of a funnel. This results in a similar structure of the grain structure as naturally sedimented soils. The sand is poured into the hopper by means of a shovel. With the help of the funnel, the sand is trickled into the mould. By varying two different sizes (1. outlet diameter of the nozzle below the hopper and 2. drop height) and their combinations, a desired relative density can be achieved. During the paving process, the hopper is continuously moved in the horizontal direction to always ensure an approximately flat surface of the specimen. The drop height is kept as constant as possible during the paving process (Fig. 5a). The sand is trickled to about 3 mm above the top of the mould. After the trickling process, the surface of the specimen is carefully scraped with a ruler.

By using the MT-method according to Ladd (1978)^33^ the specimen is mounted in a certain number of layers using a selected degree of undercompaction. In the case of the 200 mm high specimens, 8 layers and a degree of undercompaction of *U* = 10 % were chosen. The wet ramming procedure is performed by means of a height-adjustable rammer (Fig. 5b). The tamper used consists of the tamped weight (diameter 50 mm, corresponding to half the sample diameter), the rod connected to it and a guide of this rod in a crossbar. A PVC ring is placed on the trickle protection as a tamper’s traverse. The crossbeam can be freely moved horizontally on the upper edge of the PVC ring. A movable and fixable spacer is used to set the height to which the specimen is to be pressed when paving a particular layer. The height of the specimen after stamping a layer is determined by taking into account the specified degree of undercompaction. Before stamping each layer, the tamper is adjusted to the calculated layer thickness and place the subsample intended for the layer into the specimen former. This is distributed evenly over the sample cross-section and then compacted with the tamper. In order to achieve the most homogeneous distribution of the layer density, the tamper should be moved continuously in clock or counterclockwise direction. Then, the pores of the samples were first flowed of CO$_2$ before fully saturated with water to allow a precise measurement of volume changes. These were done by means of a differential pressure transducer DPT with 10 mbar fullscale and a resolution of 65 $\mu$m that was connected to a pipette system of 1 m length using a back pressure of 500 kPa leading to B-values higher than 0.98.

## Methodolody and test results

**Figure 6:**
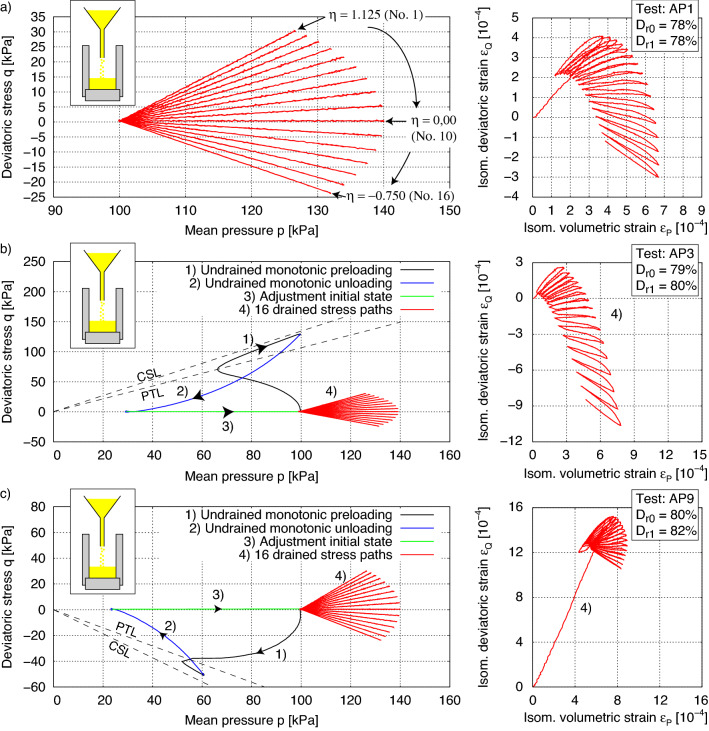
Test results on KFS with a critical friction angle $\varphi _c$ = 33.1$^\circ$: (**a**) without preshearing history of Test AP1 as reference test, (**b**) preshearing history in compression area of Test AP3 with mobilized friction angle $\varphi _{\text {mob}}$ = 32.4$^\circ$ and (**c**) preshearing history in extension area of Test AP9 with mobilized friction angle $\varphi _\text {mob}$ = 29.1$^\circ$. $D_{r0}$ after sample preparation (air pluviation) and $D_{r1}$ at start of drained stress paths.

**Figure 7:**
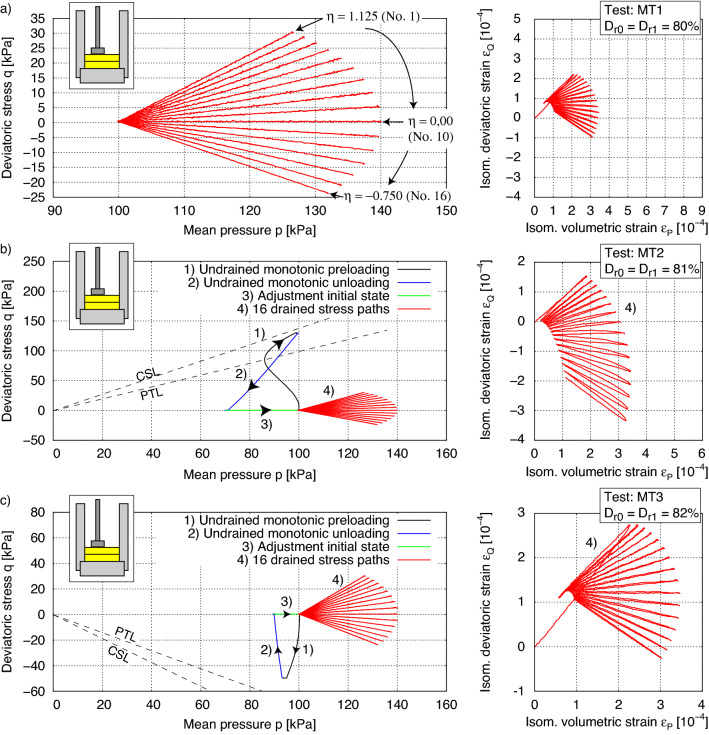
Test results on KFS with a critical friction angle $\varphi _c$ = 33.1$^\circ$: (**a**) without preshearing history of Test MT1 as reference test, (**b**) preshearing history in compression area of Test MT2 with mobilized friction angle $\varphi _\text {mob}$ = 32.7$^\circ$ and (**c**) preshearing history in extension area of Test MT3 with mobilized friction angle $\varphi _\text {mob}$ = 16.9$^\circ$. $D_{r0}$ after sample preparation (moist tamping) and $D_{r1}$ at start of drained stress paths.

In addition to the undrained cyclic tests by Ishihara and Okada^2^, extended experiments with undrained and drained stress paths considering small and large preshearing histories were performed in the presented cyclic triaxial device. In all tests, the same drained cyclic loading paths were applied, but different preshearing histories were taken into account. The dense samples of KFS were prepared with an equal initial density ($D_{r0} = (e_{\max } - e)/(e_{\max } - e_{\min }) \approx 0.8$) as specified in Tables 1 and 2. Figure 6 shows the results of selected experiments using AP-method, while Fig. 7 presents corresponding results for the MT-method. All initial and preshearing conditions of the tests are summarized in Tables 1 and 2.

**Table 1:** Testing program with samples prepared by dry air pluviation (AP).

Test no.	*p* (kPa)	*q* (kPa)	$D_{r0}$ (%)	$D_{r1}$ (%)
AP1	0	0	78	78
AP2	77.4	83.0	80	80
AP3	99.5	128.5	79	80
AP4	346.8	495.6	78	79
AP5	612.2	903.6	79	80
AP7	89.5	−28.5	82	83
AP8	82.2	−39.6	80	81
AP9	60.5	−50.4	80	82

AP1 as reference test. AP2–5 with undrained preshearing in triaxial compression and AP7–9 in triaxial extension.

**Table 2:** Testing program with samples prepared by moist tamping (MT).

Test no.	*p* (kPa)	*q* (kPa)	$D_{r0}$ (%)	$D_{r1}$ (%)
MT1	0	0	80	80
MT2	99.1	129.9	81	81
MT3	93.7	−49.6	82	82

MT1 as reference test. MT2 with undrained preshearing in triaxial compression and MT3 in triaxial extension.

The AP1 test without an undrained preshearing history represents the reference test (Fig. 6a). Starting from an isotropic stress state with an initial mean effective stress $p_0$ = 100 kPa, drained *p*-*q* stress paths of length $l_{pq} = \sqrt{p^2+q^2}=40$ kPa were investigated under 16 different stress ratios $\eta$ (= *q*/*p*) in the compression and extension area (Fig. 6a). One cycle was applied for each stress ratio. The stress ratios $\eta$ were varied in steps of $\Delta \eta$ = 0.125 ($\eta = 1.125; 1.00; 0.875; \dots ; -0.625$ and $-0.750$). The first stress path with stress ratio $\eta$ = 1.125 represents a loading and unloading of the specimen. Each stress path with stress ratio $\eta _i$ (loading) is therefore followed by another stress path $-\eta _i$ (unloading), until the initial stress state *p* = 100 kPa and *q* = 0 kPa is reached. After that, the next stress path is applied with $\eta _{i+1}$. In an analogous manner, the other 15 stress paths were applied up to the 16th stress path with $\eta = -0.750$. This was done by a preprogrammed sequence of loading and unloading linear ramps. The measured strain paths are shown in Fig. 6a on the right. The initial density $D_{r1}$ before the start of the first stress path ($\varepsilon _P$ = $\varepsilon _Q$ = 0) remained almost unchanged compared to the relative desity after the preparation process, i.e. $D_{r1}$ = $D_{r0}$ = 78%. To investigate the influence of a preshearing history on the material behaviour during subsequent cyclic loading, additional specimens were preloaded under undrained conditions prior to application of the drained loading paths in either the compression or extension area, as specified in Table 1.

In the AP3 test with a preshearing in triaxial compression, an increase in deviatoric stress to *q* = 128.5 kPa was first performed along the critical state line in *p*–*q* space, see Fig. 6b. It was followed by undrained unloading, opening the drainage, and adjustment of the initial pre-cyclic stress state $p = 100$ kPa, $q = 0$ kPa. The plot on the right side of Fig. 6b shows the strain paths obtained during the subsequent drained stress cycles. The obtained deviatoric strain components are approximately 3 times larger in the opposite direction of preshearing than in the reference test without preshearing (Fig. 6a), which will be addressed in the next section along with the discussion on constitutive models performance. Based on the effective stress path during undrained preshearing in Fig. 6b, the friction angle at the phase transformation line (PTL) can be determined to $\varphi _{PTL} = \arcsin (3\cdot \eta _{PTL}/(6+\eta _{PTL})) = {26.78^\circ }$. After undrained preshearing in the extension area (Fig. 6c), application of the first drained stress path with stress ratio $\eta$ = 1.125 showed much larger deformation than for the subsequent loading and unloading paths. A comparison of the $\varepsilon _P$-$\varepsilon _Q$ paths of Fig. 6b,c from the constitutive point of view suggests a rotation of the yield surface due to the undrained preshearing history and hardening along preshearing direction.

**Figure 8:**
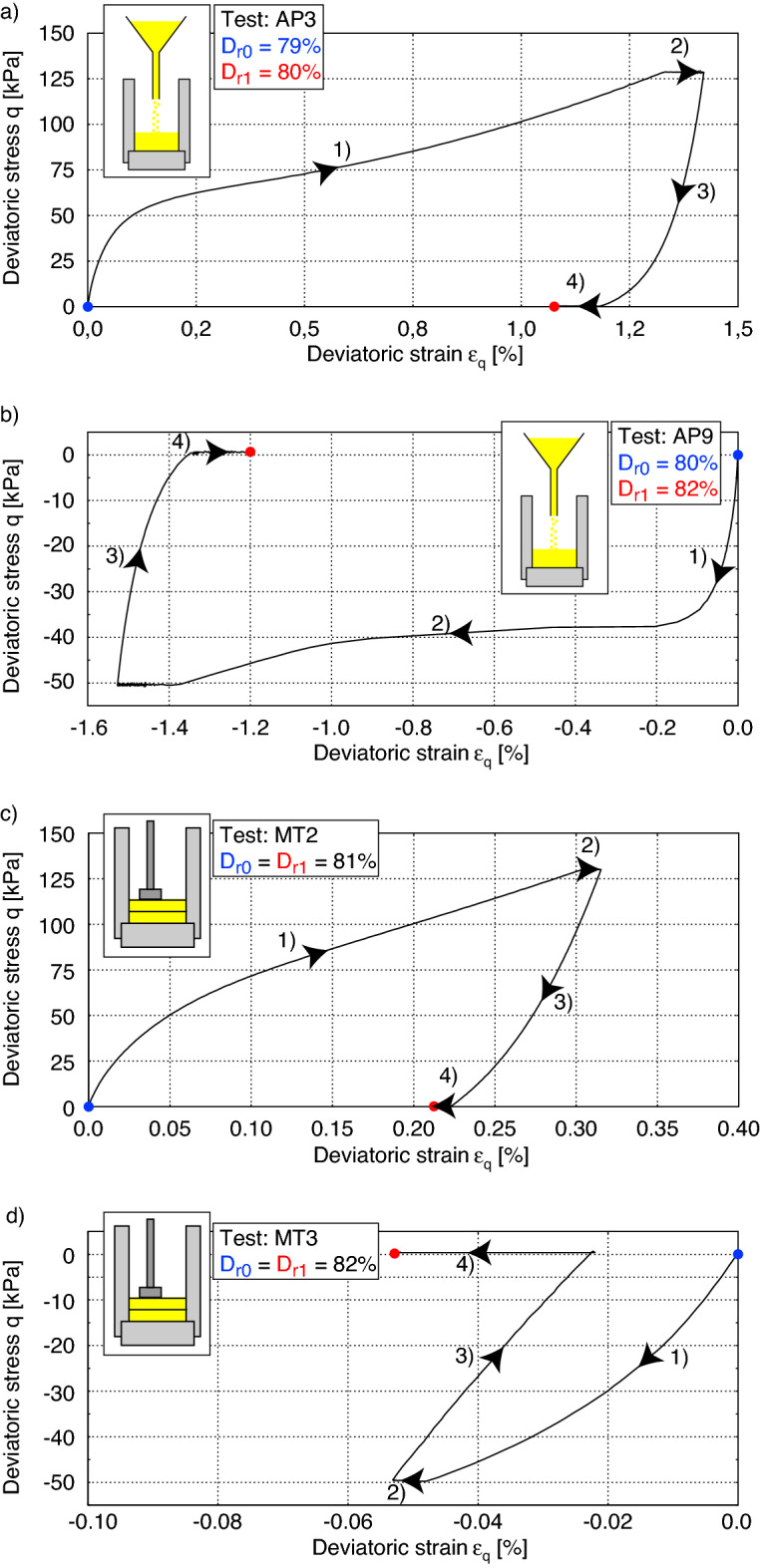
*q*–$\varepsilon _q$ diagrams during the preloading paths of tests AP3, AP9, MT2 and MT3 from the Figs. 6b,c and 7b,c.

**Figure 9:**
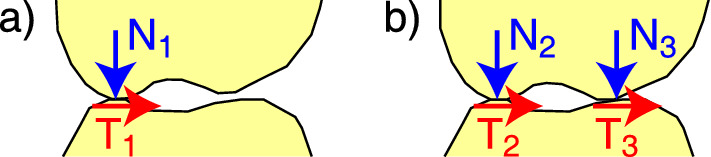
(**a**) One grain contact and (**b**) two grain contacts.

Figure 7 shows the same experiments as in Fig. 6, but on specimens prepared by the wet MP-method (Table 2). Thereby the influence of preshearing is less pronounced than in Fig. 6 for the AP method. Compared to the AP-method, the specimens prepared with the MP-method show a lower tendency to contractive behaviour during the undrained loading and unloading phases. This can be explained by the fact that more grain contacts (Fig. 9) are induced by the MT technique at the same density. A large portion of the preloading was effectively pre-induced by the introduced energy when the sample was prepared. Hence, a preconditioning of the grain structure takes place by the applied energy, which is much greater than with AP. The friction angle at the PTL is here determined to be $\varphi _{PTL}$ = 22.6$^\circ$, which is lower than the one for samples prepared by AP. This feature allows the MT sample more dilatancy and consequently a higher resistance to liquefaction. The *q*-$\varepsilon _q$ paths during the preloading paths presented in Figs. 6b,c and 7b,c are shown in the Fig. 8. The change in void ratio in the MT-tests was marginal and lead to equal relative densities, while in AP3 and AP9 a difference between $D_{r0}$ and $D_{r1}$ of 1 and 2% was encountered, respectively. Consequently, Fig. 8b shows a comparatively larger deviatoric strain for AP9 with a preshearing in the extension range.

Along with this stand the observations that uncompacted samples prepared by air pluviation typically have the lowest undrained cyclic strength, whereas samples made by moist tamping have been shown to endure more cycles until liquefaction^13,17,19,20,35,36^. Ladd^37,38^ reported, that the differences between the results depend on (1) differences in grain and interparticle contact orientations, (2) different variations of void ratio (dry unit weight) within specimens and (3) segregation of particles. The MT technique introduces an anisotropic behaviour which does not ensure completely homogeneous conditions. In general, it can be said that a higher relative density results in more grain contacts. In a comparison of the AP and MT preparation methods, the MT technique leads to more grain contacts and thus to a lower contracting behaviour under undrained conditions. Mulilis et al.^39^ showed, that the preferred orientation of tangential planes at contacts for samples compacted by AP with 11$^\circ$ are lower than by MT with 48$^\circ$. Figure 9 shows schematically the arrangement and force transmission of one or two grain contacts. With otherwise identical grains, the MT technique produces more grain contacts in the course of tamping, analogous to Fig. 9b.

Nevertheless, there is a novel study presented by^36^ investigating the microscopic influence of sample preparation method on Toyoura sand using an image-analysis-based technique. There has been stated that the sand samples prepared by AP and MT methods can be reasonably assumed to be transversely isotropic, with the vertical direction as the axis of symmetry. In the vertical plane, the AP specimens possessed a pronounced inherent anisotropy, whereas the MT samples tended to be more isotropic. This distinct behaviour to the one described previously may be attributed to the shape of the grains which is an intrinsic property^40^.

## Comparative simulations with four advanced constitutive models

The aim of this research paper is to assess the prediction quality of three established as well as one novel constitutive model with the special attention to preshearing history of sands using small and large strain amplitudes. Constitutive models that can describe more complicated stress paths, such as a cyclic loading with a number of cycles $N \le 100$, in addition to monotonic loading are investigated. Input parameters for KFS were already determined based on a detailed investigation of existing and well-documented laboratory experiments e.g.^41–43^. As representatives of the advanced and at the same time (comparatively) widely used material models, the hypoplasticity^44^ with intergranular strain^45^ (Hypo+IGS), the intergranular strain anisotropy model (ISA)^46^, and the elastoplastic model SaniSand^47^ are considered. Recent developments are represented by the model with historiotropic yield surface (so-called Hypo+YS)^42^. A brief summary of the equations and main properties of each model is given in Appendix A.

Of particular interest here is the prediction of the presented tests with various preshearing paths on KFS samples. However, due to the limited variation of boundary conditions, these tests are unsuitable for determining all parameters of a constitutive model. Wichtmann et al.^41^ documents a numerical study in which the extensive and well-documented database on KFS from^48–50^ has been used to calibrate and inspect SaniSand, Hypo+IGS and ISA, while in^42^ it has been done for Hypo+YS. This paper uses the parameters obtained and validated in these studies and listed in Table 3, 4 and 5.

For each constitutive model, a user defined material routine (UMAT) by A. Niemunis (Hypo+IGS), M. Tafili (SaniSand and ISA) and C.E. Grandas Tavera (Hypo+YS) was available. The element test simulations were performed with the software *incremental driver* developed by A. Niemunis^51^. The classic “elastic predictor” scheme^52^ has been followed to perform the numerical implementation of SaniSand and ISA. A substepping scheme with small strain increments has been implemented to guarantee numerical convergence in each subroutine.

The hypoplastic model with intergranular strain requires the calibration of eight parameters for monotonic loading and additional five parameters for the intergranular strain, hence for cyclic loading, as listed in Table 3. Hereby, the parameters calibrated in^41^ using various drained and undrained monotonic and cyclic triaxial tests as well as oedometric tests with varying initial conditions were used.

**Table 3:** Material parameters of Hypo+IGS for KFS from^41^.

$\varphi _c$ (–)	$e_{i0}$ (–)	$e_{c0}$ (–)	$e_{d0}$ (–)	$h_{s}$ (MPa])	*n* (–)	
33.1$^\circ$	1.212	1.054	0.677	4000	0.27	

$\alpha$ (–)	$\beta$ (–)	*R* (–)	$m_R$ (–)	$m_T$ (–)	$\beta _R$ (–)	$\chi$ (–)
0.14	2.5	10$^{-4}$	2.2	1.1	0.1	5.5

The ISA model requires the calibration of 12 parameters involved in the description of the mechanical behaviour of sands under monotonic loading and additionally 6 parameters defining the intergranular strain anisotropy. The parameters calibrated in^41^ for KFS are employed and presented in Table 4. Thereby, the parameter $r_F$ accounting for the inherent fabric arising for example from the sample preparation method has been varied from $r_F=1.6$ for AP technique to $r_F=0.0$ for the MP method as explained in the Appendix A.2.

**Table 4:** Material parameters of ISA model for KFS from^41^.

$e_{i0}$ (–)	$\lambda _i$ (–)	$n_{pi}$ (–)	$n_e$ (–)	$\nu$ (–)	$e_{c0}$ (–)
1.21	0.0045	0.8	3.2	0.35	1.067

$\lambda _c$ (–)	$n_{pc}$ (–)	$M_c$ (–)	*c* (–)	$n_d$ (–)	$f_{b0}$ (–)
0.00573	0.68	1.34	0.7	0.5	1.8

*R* (–)	$m_R$ (–)	$\beta$ (–)	$\chi _h$ (–)	$c_{z}$ (–)	$r_F$ (–)
10$^{-4}$	1.7	0.1	11	50,000	1.6 / 0.0

The determination of in total 15 material parameters, as listed in Table 5, is required for the SaniSand model. Therefore undrained monotonic and cyclic triaxial tests, oedometric tests as well as drained monotonic tests are necessary. These parameters are taken from^41^ as well, see Table 5.

**Table 5:** Material parameters of SaniSand for KFS from^41^.

$e_{0}$ (–)	$\lambda$ (–)	$\xi$ (–)	$M_c$ (–)	$M_e$ (–)
1.103	0.122	0.205	1.34	0.938

*m* (–)	$G_0$ (–)	$\nu$ (–)	$h_0$ (–)	$c_h$ (–)
0.05	150	0.05	10.5	0.75

$n_b$ (–)	$A_0$ (–)	$n_d$ (–)	$z_{\max }$ (–)	$c_z$ (–)
1.2	0.9	2	20	10,000

The recently developed constitutive model with a historiotropic yield surface^42^ requires the calibration of in total 16 parameters consisting of 3 parameters for the hyperelastic stiffness tensor, 4 for the critical state, 3 for the limiting compression curve, two for the dilatancy and 4 parameters for the yield surface involving oedometric tests as well as monotonic and cyclic triaxial experiments. The parameters used for the following simulations are taken from^42^ as listed in Table 6.

**Table 6:** Material parameters of Hypo+YS for KFS from^42^.

$\alpha$ (–)	*n* (–)	*c* (–)	$\varphi _c$ (–)
0.1	0.677	0.001096	33.1$^\circ$

$e_{c0}$ (–)	$n_{Bc}$ (–)	$h_{sc}$ (MPa)	$c_2$ (–)
1.054	0.27	4000	50

$n_{Peak}$ (–)	$n_{YD}$ (–)	$e_{i0}$ (–)	$c_b$ (–)
2	1	1.1	0.2

$n_{PTL}$ (–)	$n_0$ (–)	$n_{Bi}$ (–)	$h_{si}$ (MPa)
1	4	0.48	8400

### Cyclic behaviour of KFS subjected to small and large preshearing

The drained cyclic tests with various preshearing histories were carried out at an initial mean pressure $p_0$ = 100 kPa. This stress condition was first achieved by isotropic consolidation of fully saturated samples. Some of them were subsequently subjected to various preshearing paths, either in compression or extension regime. Therefore, the phase transformation line (PTL) is considered as a boundary line separating two different domains in the stress space: one where the sample develops large strains and another where it develops small strains as indicated by Ishihara and Okada^2^ and described in “Introduction”.

All simulations were conducted with the initial confining pressure $\sigma _1 = \sigma _2 = \sigma _3$ = 100 kPa considering all subsequent preshearing histories. The initial void ratio is calculated following the relation:

1 $$\begin{aligned} e_0=e_{\max }-D_{r0}\left( e_{\max }-e_{\min }\right) \end{aligned}$$

which for the dense samples tested herein (see Tables 1 and 2) renders $e_0=0.75-0.76$. An initially fully-mobilized intergranular strain under isotropic direction i.e. $\textbf{h}=-R/\sqrt{3} ~ \textbf{1}$ (Hypo+IGS and ISA) was assumed. The intergranular back stress tensor was then assumed as half the intergranular strain i.e. $\textbf{c}=-R/(2\sqrt{3}) ~\textbf{1}$ (ISA) and the back stress tensor equals to the initial stress state ${\varvec{\sigma }}_B={\varvec{\sigma }}_0$ (SaniSand and Hypo+YS). Each loading step was performed using proportional paths with 1000 increments and Roscoe variable controls with $\Delta q$ and $\Delta p$ or $\Delta \varepsilon _v$ corresponding to the amplitudes and boundaries specified in “Methodolody and test results”. Other shear strains were held constant $\Delta \gamma _{12}=\Delta \gamma _{23}=0$.

**Figure 10:**
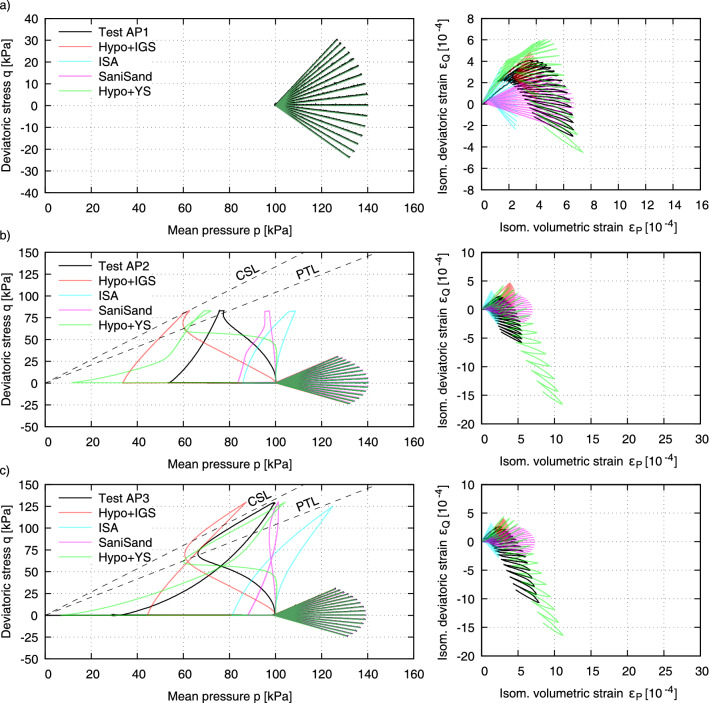
Simulations of tests AP1, AP2 (small undrained preshearing) and AP3 (large undrained preshearing in triaxial compression).

**Figure 11:**
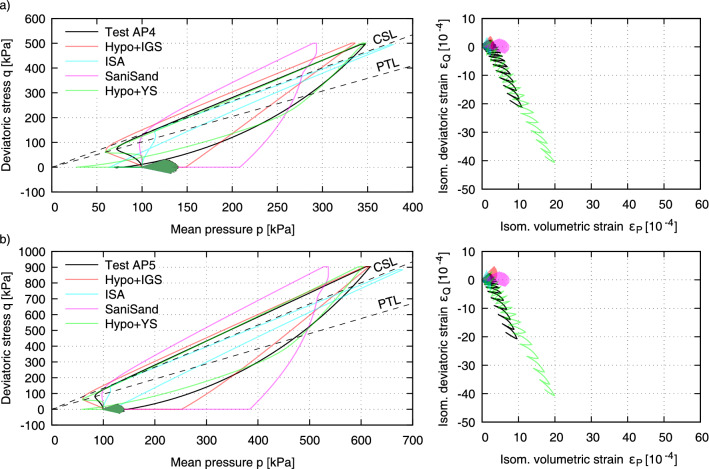
Simulations of tests AP4 (large undrained preshearing along CSL in triaxial compression) and AP5 (large undrained preshearing along CSL in triaxial compression).

Figure 10 presents the comparison between the experiments AP1 to AP3 and the simulations with the four selected constitutive models. In AP1 the drained cyclic paths commenced after isotropic consolidation (Fig. 10a). Hypo+IGS and SaniSand show a nearly symmetrical strain path with respect to the isometric volumetric strain, while Hypo+YS and to some extent also ISA follow the experimental evidence with a slightly higher overall deviatoric strain in triaxial compression. It can be recognized that both the volumetric and deviatoric strains in the first drained cycle are higher than those during the subsequent cycles. In view of the fact that the sample was previously subjected only to virgin isotropic loading, the first drained cycle in triaxial compression indicated that the sample is partly sustaining plastic strains in the course of applying shear stresses for the first time. This behaviour is well reproduced by Hypo+YS.

The AP2 sample (Fig. 10b) was subjected to a small undrained preshearing in triaxial compression, which ended slightly below the PTL was reached, while in AP3 (Fig. 10c) a large shearing between PTL and CSL in triaxial compression was applied before the drained cyclic loading took place. In both cases the experiments evidence a stiffer response of the samples on the triaxial side of the preshearing, and softer on the opposite side, hence the samples show significantly more strain accumulation in triaxial extension. While the volumetric strain in AP1, AP2 and AP3 are nearly the same, the deviatoric strain in AP2 and AP3 is nearly 2.5 and 5 times larger than in AP1, respectively. Hence, the effect of the magnitude and direction of undrained preshearing on the strain accumulation and subsequently on the liquefaction resistance of sand is essential. This effect is amplified in Fig. 11 in the case of AP4 and AP5 with an even larger preshearing to a deviatoric stress $q \approx 500$ kPa and $q \approx 900$ kPa, respectively. In both cases the deviatoric strain is 10 times larger than in AP1. Hence, two observations can be made. First, with larger preshearing in one direction the sample gets softer in the opposite direction. Secondly, after approaching the CSL, subsequent increments of the preshearing amplitude have no further influence on the strain accumulation; a threshold value is reached. Among the herein investigated constitutive models only Hypo+YS is capable of predicting this behaviour, in particular the accumulation of strain due to the fan-applied stress cycles as well as the influence of the undrained preshearing history. Due to the anisotropic back stress tensor ${\varvec{\sigma }}_B$ (see Appendix) in conjunction with the historiotropic surface, which besides the recent loading history stores the “elder” preshearing history, thus “small and large preshearing” as well, the model is able to capture the influence of small and large preshearing history on the strain accumulation of sand.

All other models generally predict the material behaviour to be too stiff. Strains during initial loading within the first half of the first cycle turn out to be too low. Also, the secant stiffness during fan-shaped stress cycles is underestimated. Unloading and reloading follow the same strain path, and there is virtually no accumulation of strain. As shown in^41^ these models perform well in cyclic tests with precompression.

**Figure 12:**
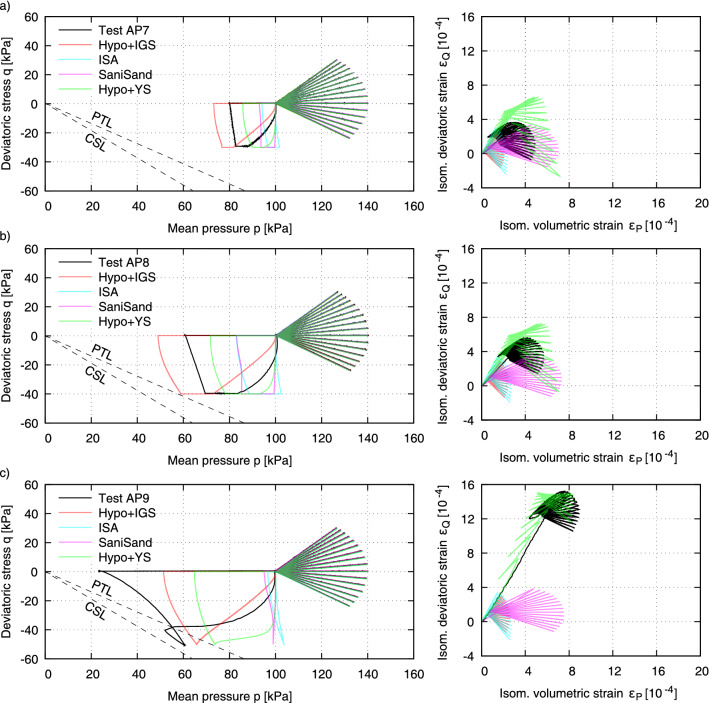
Simulations of tests AP7 (small undrained preshearing in triaxial extension), AP8 (small undrained preshearing in triaxial extension) and AP9 (large undrained preshearing in triaxial extension).

Figure 12 shows simulations of the tests AP7 and AP8 with small undrained preshearing in triaxial extension and AP9 with large undrained preshearing in triaxial extension. These tests verify the findings of the experiments AP1 to AP5 with preshearing in triaxial compression. Due to the preshearing in triaxial extension, the response of the material is here softer in triaxial compression, so that the accumulation takes place in the later direction as well and is rendered larger the larger the preshearing occured. The experimental behaviour can be reproduced only with Hypo+YS satisfactorily, while the other models show negligible influence of undrained preshearing on the subsequent strain accumulation due to drained cycles in both triaxial compression and triaxial extension.

**Figure 13:**
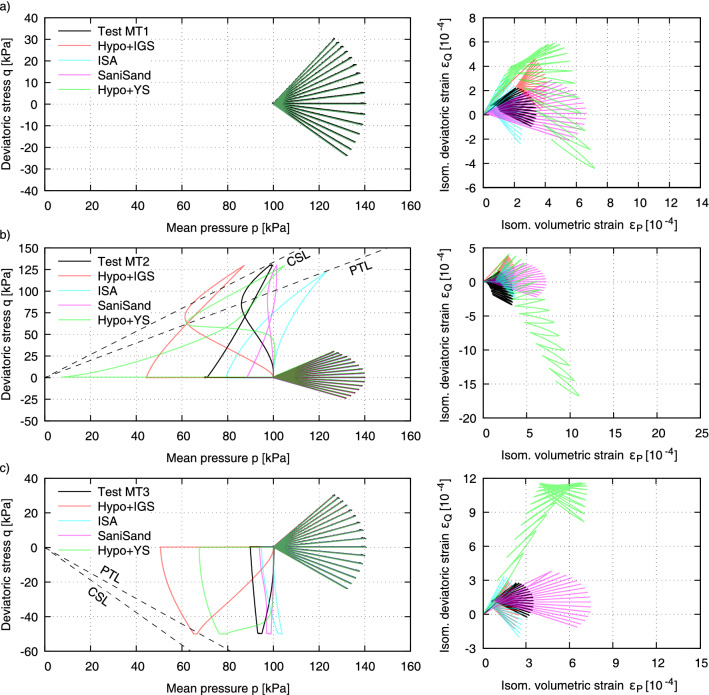
Simulations of tests with moist tamping: MT1, MT2 (large undrained preshearing in triaxial compression) and MT3 (small undrained preshearing in triaxial extension).

Some of the tests were repeated for samples prepared by moist tamping and are shown in Fig. 13: MT1 without preshearing, MT2 with large undrained preshearing in triaxial compression and MT3 with small undrained preshearing in triaxial extension. The influence of preshearing on the strain accumulation of samples prepared by this technique is less pronounced as discussed also in “Methodolody and test results”. Hypo+IGS, SaniSand and Hypo+YS show equal results to those for samples prepared by air pluviation and are thus not able to reproduce the influence of different sample preparation techniques on the mechanical behaviour of sand with the same parameter set. However, this was expected due to the fact that the same initialization procedure as for AP samples was used. The additional energy supply by the MP method is thus not reflected in the internal state variables and further research in the microscopic point of view is required for this purpose. Due to the fact that Hypo+IGS, ISA and SaniSand show a marginal influence of undrained preshearing on the strain accumulation, they provide a better agreement with the experimental results of samples prepared by MT than Hypo+YS. Additionally, the ISA parameter $r F=0.0$ was included for these simulations in order to take into account how the sample preparation procedure affected the evolution of the sand’s fabric. Therefore, when contrasting the two preparation strategies, the ISA model provides a better agreement with the experimental results. Other methods that introduce an additional tensorial state variable, whose initialization takes into consideration the contact orientations between the grains as a result of sample preparation, such as those in^36,53,54^, may be adopted in the future. Hence, without introducing the variables associated with inherent fabric a separate set of model parameters for each sample preparation method is needed for Hypo+IGS, SaniSand and Hypo+YS.

### Cyclic behaviour of Fuji River Sand subjected to large preshear

As described in the introduction, the motivation for this work was among others the findings of Ishihara and Okada^2^ where it was discovered that samples subjected to large preshear on one side of triaxial loading, compression or extension, became stiffer on that side, but softer on the other side. This behaviour is proved with the experiments in this paper also for cyclic drained post-loading as well. To investigate the models performance for undrained cyclic loading, hence also its applicability for earthquake-similar loading conditions after a large preshearing, the test presented in Fig. 2^2^ is simulated in the following. In this test, the sample was first subjected to approximately 6 cycles with $q^{{\text{ ampl }}}=0.4$ kg/cm$^2$ and then to a large deviatoric stress $q=1.1$ kg/cm$^2$ in triaxial compression. Then the sample was reconsolidated and again subjected to the cyclic deviator stress $q^{{\text{ ampl }}}=0.4$ kg/cm$^2$. At the end of first cyclic loading the mean effective stress reduced to its half value hence a pore water pressure as much as 50% of the initial confining pressure had been developed, while at the end of large preshearing it increased to 80%. In the second cyclic loading, the stiffness in the triaxial compression part was higher compared to the first cyclic loading. Subsequently, shearing in triaxial extension lead to a pore water pressure buildup of about 80% of the initial confining pressure in the very first cycle. In the first cyclic loading it amounted to approximately 25% in the very first cycle, eventhough the initial void ratio was higher. The large difference in the soil behaviour between the triaxial compression and extension can be attributed to the fact that the sample has been subjected to large preshearing in triaxial compression in the first phase of cyclic loading, which is a kind of preconditioning and thereby making the sample difficult to deform further in that direction as was proven in the experimental part of this paper as well.

**Figure 14:**
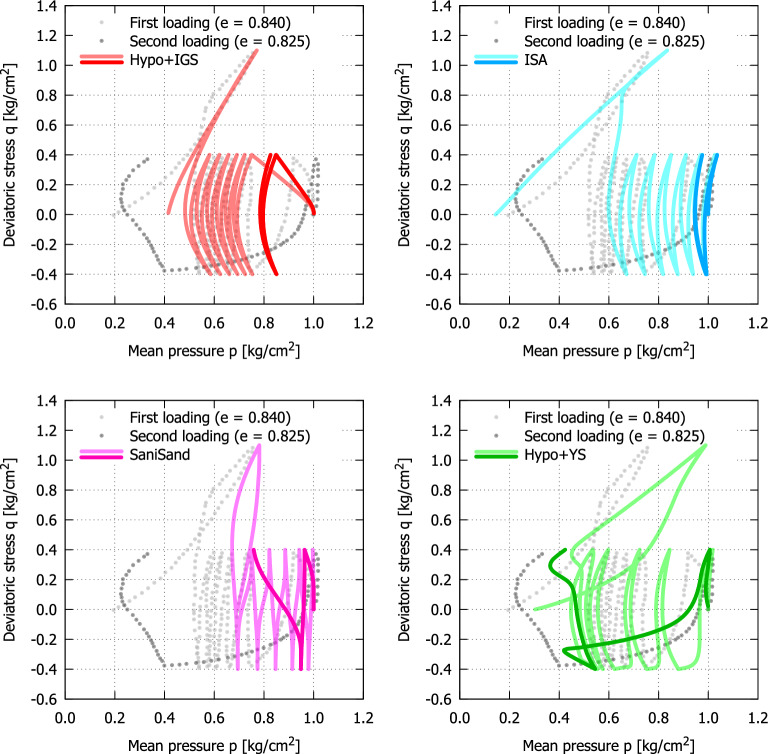
Behaviour of sand subjected to large preshear. Experiment conducted on Fuji River Sand^2^, simulations on KFS for qualitative comparison (light colors represent first loading, dark colors represent second loading).

The simulations with the models are conducted with the parameters of KFS used in the previous section, thus no recalibration of the parameters with the data provided in^2^ was undertaken because of two reasons. First, the aim of this paper is not the calibration of the models but their qualitative response to small and large preshearing. Second, in^2^ 7 cyclic tests without providing the raw data or the complete stress-strain paths are presented. A calibration of the monotonic parameters of the models based on those tests is not likely, and then no validation of the performance of the models would be possible. Hence, in order to obtain the qualitative response of the models based on parameters calibrated on an extensive database, the parameters of KFS are used. The initial void ratio was chosen considering the initial relative density of the experiment. All other state variables were initialized as pointed out in “Cyclic behaviour of KFS subjected to small and large preshearing”. Hypo+IGS as well as ISA show a stiffer response in the second cyclic loading than in the first one, eventhough the large preshearing was well modelled (see Fig. 14). The SaniSand model follows the observations of Hypo+IGS and ISA, proceeding with a softer response in the last part (triaxial compression) of the second cyclic loading, which is not in concordance with the tendency of the experiment. Solely Hypo+YS represents the material behaviour in a good agreement to the one obtained in the test. Hence the model describes well the influence of small and large preshearing on the material behaviour, making it an appropriate candidate for finite element analysis of geotechnical issues associated with earthquake hazards among others.

## Conclusion

Triaxial tests with a novel combination of undrained preshearing and drained stress paths by controlling the axial and radial stresses (coupling between mean and deviator stress) have shown that the soil structure (samples prepared by dry air pluviation or moist tamping of KFS) as well as the (magnitude of strain or stress) preshearing history have a significant influence on the soil behaviour, in particular by dealing with cyclic loading. The experiments evidence a stiffer response of the samples in the direction of preshearing, and softer on the opposite side. After approaching the CSL, the increasing preshearing amplitude has no further influence on the strain accumulation; a thresshold value is likely to be reached. In contrast, the influence of preshearing on the strain accumulation of samples prepared by moist tamping was found negligible. This effect may be attributed to the fact that more grain contacts or a precondition to cyclic loading are present in the MT-samples due to the preparation method and thus less possibility for rearrangement of the grains (induced anisotropy) during preshearing is expected.

Four advanced constitutive models are inspected by using well documented material parameters of KFS from the literature. Solely Hypo+YS was able to represent the influence of small and large preshearing amplitudes on the soil behaviour in a good agreement to the experiments. In contrast, Hypo+IGS, ISA and SaniSand have shown some fundamental disadvantages in the reproduction of large preshearing effects. Finally, the undrained cyclic triaxial test with large preshearing from^2^ was simulated with the selected models and it emerged that, only Hypo+YS can reproduce the strong reduction in the number of cycles to liquefaction due to a large preshearing.

## Data Availability

The datasets generalized and analysed during the current study are available from the corresponding author on reasonable request.

## A. Constitutive equations

### A.1 Hypoplasticity with intergranular strain (Hypo+IGS)

The hypoplastic model for sand proposed in 1996^44^ is herein used in conjunction with the intergranular strain porposed in 1997^45^. The new state variable $\textbf{h}$ named intergranular strain has been introduced for hypoplastic models in order to improve their performance in the range of small load cycles.

In general, the constitutive equation of Hypo+IGS relates the objective effective stress rate ${\mathring{{\varvec{\sigma }}}}$ with the strain rate ${\dot{{\varvec{\varepsilon }}}}$:

$$\begin{aligned} {\mathring{{\varvec{\sigma }}}} = \textsf{M}({{\varvec{\sigma }}},{\mathop {\dot{{\varvec{\varepsilon }}}}\limits ^{\rightarrow }},\textbf{h},e) : {\dot{{\varvec{\varepsilon }}}} \end{aligned}$$

wherein $\textsf{M}$ is a fourth order tensor representing the tangential stiffness. It is calculated from the barotropic and pyknotropic hypoplastic tensors ${\textsf{L}}({\varvec{\sigma }},e)$ and $\textbf{N}({\varvec{\sigma }},e)$ which are suitably increased, depending on the loading direction and the size of the evolved intergranular strain. For example, for monotonic deformation with $\dot{{\varvec{\varepsilon }}}\propto {\mathop {\textbf{h}}\limits ^{\rightarrow }}$ the hypoplastic equation:

2 $$\begin{aligned} {\mathring{{\varvec{\sigma }}}} = \left( {\textsf{L}}+ \textbf{N}\frac{{\dot{{\varvec{\varepsilon }}}}}{\Vert {\dot{{\varvec{\varepsilon }}}}\Vert } \right) : {\dot{{\varvec{\varepsilon }}}} \end{aligned}$$

is recovered. For reversed deformation, i.e. $\dot{{\varvec{\varepsilon }}}\propto -{\mathop {\textbf{h}}\limits ^{\rightarrow }}$^24^ the stiffness is increased by the material parameter $m_R$ and the nonlinear part of the hypoplastic equation is deactivated, hence ${\textsf{M}}=m_R{\textsf{L}}$, ${\textsf{L}}$ being the elastic fourth order tangent stiffness tensor. Finally, under neutral strain rate, i.e. $\dot{{\varvec{\varepsilon }}}\perp {\mathop {\textbf{h}}\limits ^{\rightarrow }}$ a slightly increased stiffness is obtained using the parameter $m_R\ge m_T\ge 1$, i.e. ${\textsf{M}}=m_T{\textsf{L}}$.

For further details on the equations of Hypo+IGS, the attention of the reader is drawn to^24,44,45^.

### A.2 Intergranular strain anisotropy model (ISA)

The intergranular strain anisotropy (ISA) model is proposed in^55^ by extending and reformulating the intergranular strain of^45^. The elasto-plastic formulation of the intergranular strain coupled with a plastic mechanical response of the model under fully mobilized intergranular strain renders the model elasto-hypoplastic. This is achieved through a yield and a bounding surface within the intergranular strain space:

$$\begin{aligned} F_H = \quad \Vert \textbf{h}-\textbf{c}\Vert -R/2 \quad \text{(yield } \text{ surface), }\\ F_{Hb}=\quad \Vert \textbf{h}\Vert -R \quad \text{(bounding } \text{ surface), } \end{aligned}$$

wherein the second order tensors $\textbf{h}$ and $\textbf{c}$ denote the intergranular strain and the back intergranular strain, respectively. The size of the yield surface is governed by its radius, the material parameter *R* at which only slight degradation of the shear modulus is allowed, i.e. the ratio $G/G_{\max }\approx$ const.

The constitutive equation for the mechanical behaviour interrelates the stress rate $\dot{{\varvec{\sigma }}}$ with the strain rate $\dot{{\varvec{\varepsilon }}}$ via the hypoplastic tangential stiffness ${\textsf{E}}$:

3 $$\begin{aligned} \dot{{\varvec{\sigma }}}={\textsf{E}}:\left( \dot{{\varvec{\varepsilon }}}-\dot{{\varvec{\varepsilon }}}^p\right) \end{aligned}$$

with the plastic strain rate dependent on $\dot{{\varvec{\varepsilon }}}^p({\varvec{\sigma }},\dot{{\varvec{\varepsilon }}},\textbf{h},e)$. Inside the intergranular yield surface $\Vert \textbf{h}\Vert <R$ the response of the model is elastic, i.e. $\dot{{\varvec{\varepsilon }}}^p=0$ in Eq. (3). During the kinematic hardening of the yield surface towards the bounding surface the so-called transition regime takes plase, hence slight plastic strain rates are allowed. Once the intergranular strain lies on the bounding surface, any influence of it is erased and the model is in fully mobilized state turning hypoplastic. In the original publication^55^ it is claimed that a smooth transition between elastic and hypoplastic regime is achieved. This might hold for triaxial tests as shown in various works for sands^41,46,56,57^ and clays^58–61^, but not necessarily also for cyclic torsional tests as shown in^62^.

Among the models considered in this work, the ISA model is the only one which accounts for the inherent and dilatancy fabric. The inherent anisotropy depending on the sample preparation method is considered via the parameter $r_F$. Lower values of $r_F$ deliver a more dilatant response of the model. For example, if the moist tamping method is considered as the preparation method rendering the most dilative behaviour, the value $r_F=0$ delivers a good approximation, whereas for other sample preparation methods $r_F=1-3$ is recommended^55^.

### A.3 Simple anisotropic sand model (SaniSand)

The SaniSand family of models has attracted increased attention from researchers in the last decades resulting in a significant number of published models, for e.g.^47,63–66^. Lastly, the yield surface of this model was reduced even to zero and becomes identical to the stress point itself, and plastic loading occurs for any direction of the stress ratio rate on which the loading and plastic strain rate directions now depend, rendering the model incrementally non-linear^67^. Hence the model is in this version transformed to a kind of hypoplasticity. Still, the most used version is the one developed by Dafalias & Manzari in 2004^47^ and therefore it will be used in the following.

It represents a ”wedge”-type yield surface in $p-q$ space in generalized form obeying the following relationship:

$$\begin{aligned} f=\left[ \left( \textbf{s}- p\, {\varvec{\alpha }}\right) :\left( \textbf{s}- p \,{\varvec{\alpha }}\right) \right] ^{1/2}-\sqrt{2/3}\,p\,m=0 \end{aligned}$$

with the deviatoric stress tensor $\textbf{s}$, back stress ratio tensor ${\varvec{\alpha }}$ and the material parameter *m* defining the opening of the wedge. Besides these variables, a fabric-dilatancy internal tensorial variable $\textbf{z}$ to model the effect of fabric change on dilatancy is introduced into the model. The elasto-plastic (subscript ep) evolution equation of the stress then takes the following dependencies:

$$\begin{aligned} \dot{{\varvec{\sigma }}}={\textsf{E}}^{ep}({\varvec{\sigma }},{\varvec{\alpha }},\textbf{z},e,{\varvec{\varepsilon }}^p):\dot{{\varvec{\varepsilon }}} \end{aligned}$$

with the plastic strain ${\varvec{\varepsilon }}^p$. For further details on the mathematical formulation of SaniSand, the interested reader is referred to^47^.

### A.4 Hypoplasticity with historiotropic yield surface (Hypo+YS)

The model with historiotropic yield surface developed by^42^ is known also as constitutive anamnesis model for sand due to its similarity to the constitutive anamnesis model for clay (CAM)^68^. It combines the hypoplastic equation with a yield surface in the stress space^31^. The yield surface is used to describe the intensity of anelastic flow by defining the state of the soils by means of the current stress, void ratio and back-stress tensor ${\varvec{\sigma }}_B$. Nevertheless, the response of the material model within the flow surface is not elastic, instead the intensity of plastic strain rates becomes dependent on the distance to the flow surface.

The main evolution equation of the model interrelates the stress rate with the strain rate using a hypoplastic-type of formulation:

4 $$\begin{aligned} \dot{{\varvec{\sigma }}}={\textsf{E}}:\left( \dot{{\varvec{\varepsilon }}}-Y\textbf{m}\Vert \dot{{\varvec{\varepsilon }}}\Vert \right) \end{aligned}$$

wherein the fourth rank tensor ${\textsf{E}}({\varvec{\sigma }},e)$ is a hyperelastic stiffness, $Y({\varvec{\sigma }},e,{\varvec{\sigma }}_B)$ is the so called degree of nonlinearity and $\textbf{m}({\varvec{\sigma }},e,{\varvec{\sigma }}_B,{\mathop {\dot{{\varvec{\varepsilon }}^*}}\limits ^{\rightarrow }})$ is the flow rule. ${\varvec{\varepsilon }}^*$ denotes the deviatoric part of the strain tensor.

As a distinctive feature of the model, a generalization of Taylor’s dilatancy rule^69^ ensures the reproduction of strong contractancy upon reversal loading observed in experiments without the introduction of additional state variables as for instance the fabric-dilatancy tensor.

For detailed insight into the model formulation, the reader’s attention is drawn to^31^.
